# Feedbacks between protistan single-cell activity and bacterial physiological structure reinforce the predator/prey link in microbial foodwebs

**DOI:** 10.3389/fmicb.2014.00453

**Published:** 2014-09-05

**Authors:** Eva Sintes, Paul A. del Giorgio

**Affiliations:** ^1^Department of Limnology and Oceanography, University of ViennaVienna, Austria; ^2^Département des Sciences Biologiques, Université du Québec à MontréalMontréal, QC, Canada

**Keywords:** heterotrophic nanoflagellates, heterotrophic bacteria, single-cell activity, aquatic food webs, feedbacks, functional response, predator-prey link

## Abstract

The trophic interactions between bacteria and their main predators, the heterotrophic nanoflagellates (HNFs), play a key role in the structuring and functioning of aquatic microbial food webs. Grazing regulation of bacterial communities, both of biomass and community structure, have been frequently reported. Additionally, bottom-up responses of the HNF at the population level (numerical responses) have also been extensively described. However, the functional response of HNF at the single-cell level has not been well explored. In this study, we concurrently measured the physiological structure of bacterial communities and HNF single-cell activities during re-growth cultures of natural aquatic communities. We found that changes in the abundance and proportion of the preferred, highly active bacterial prey, caused by the feeding activity of their predators (HNF), induced a negative feedback effect on the single-cell activity of these HNF. These shifts in the specific cellular activity of HNF occur at a much shorter time scale than population level shifts in flagellate abundance, and offer a complementary mechanism to explain not only the tight coupling between bacteria and HNF, but also the relative constancy of bacterial abundance in aquatic ecosystems.

## INTRODUCTION

Bacteria play a key role in aquatic biogeochemical cycles (Cho and Azam, 1988). It is now clear that trophic interactions, including viral infection and grazing by unicellular protists, play a major role in regulating the overall bacterial biomass and activity in both the water column and sediments of oceans and lakes. The relative contribution of both processes to the total bacterial mortality varies depending on system trophic status (Weinbauer and Peduzzi, 1995; Weinbauer et al., 2003), oxic conditions (Weinbauer and Hofle, 1998), or the season (Personnic et al., 2009). In particular, grazing by some of the smallest eukaryotic components, the heterotrophic nanoflagellates (HNFs), has been shown to account for up to 100% of bacterial production in oceans (Vazquez-Dominguez et al., 2005; Unrein et al., 2007; Pearce et al., 2010), estuaries (Painchaud et al., 1996), and lakes (Weinbauer and Hofle, 1998; Comte et al., 2006), and it has been suggested to have a stronger effect on bacterial biomass, whereas viral lysis is considered to influence more strongly the diversity of their host populations (Pernthaler, 2005). A tight coupling between bacteria and their protozoan grazers has often been reported, both *in situ* and in experimental incubations (Rassoulzadegan and Sheldon, 1986; Weisse and Scheffelmoser, 1991; Sanders et al., 1992), which has been explained by the fact that the predator (HNF) can potentially grow at similar rates as the prey (bacteria), and therefore effectively track the changes in bacterial abundance or biomass (Fenchel, 1982c).

Grazing not only regulates bacterial biomass in oceans and freshwaters, but also profoundly influences bacterial community structure as well. Aquatic bacterial communities are extremely complex and heterogeneous, composed of many coexisting taxa with different intrinsic metabolic rates (Cottrell and Kirchman, 2003; Yokokawa and Nagata, 2005; Alonso and Pernthaler, 2006), and with a large physiologic flexibility on a community level (Baker et al., 1998; Egli, 2010). As a result, within any given bacterial community there is a continuum of physiological states and cell characteristics, which collectively determine the physiological structure of the community (del Giorgio and Gasol, 2008). Environmental conditions, including temperature (Choi et al., 1999), starvation (Lopez-Amoros et al., 1995; Reis et al., 2005), and nutrient availability (Gasol et al., 1999), influence the physiological structure of bacterioplankton assemblages. Another key determinant of the composition and physiologic structure of bacterial communities are trophic interactions. For example, viruses may influence composition by selectively infecting dominant taxa or strains (Thingstad and Lignell, 1997; Rodriguez-Brito et al., 2010; Winter et al., 2010), and also remove the most active cells (Middelboe, 2000; Weinbauer, 2004). Likewise, HNF have been shown to be highly selective (Montagnes et al., 2008), with feeding preferences based on taxonomic composition (Christoffersen et al., 1997; Jezbera et al., 2005; Gerea et al., 2013), prey morphology (Jürgens and Matz, 2002), and prey activity (Gasol et al., 1999; Gasol and del Giorgio, 2000). This selectivity may result in profound shifts in both the composition of the bacterial communities and their physiological structure (Pernthaler, 2005).

Whereas it is now clear that HNF can profoundly impact not only the total bacterial biomass and composition, but also the distribution of single-cell characteristics of natural bacteria, it is less clear whether the reverse also holds true, i.e., whether shifts in the distribution of bacterial single-cell characteristics may affect protistan single-cell activity. Most grazing studies have focused on the numerical response of HNF at the community or population level (Weisse and Scheffelmoser, 1991; Mohapatra and Fukami, 2004), and only a limited number of studies have assessed the functional response of HNF to changes in prey abundance (Boenigk et al., 2002) or prey quality (Shannon et al., 2007; Grover and Chrzanowski, 2009; Meunier et al., 2012; Simek et al., 2013), which collectively suggest that HNF may express a relatively wide range of feeding activity as a response to the availability and type of prey. This latter connection is key to our understanding of the functioning of microbial food webs, because it represents a predator-prey feedback that operates at times scales much shorter than the numerical responses that occur at the population level.

Most of the work on HNF functional responses to prey quality or structure has been carried out under highly controled conditions, using single HNF species fed specific bacterial prey, because quantification of these responses in natural, mixed HNF and bacterial assemblages has been technically very challenging, so it is unclear how these results apply to ambient HNF communities feeding on natural mixed bacterial prey. In this regard, in a previous paper (Sintes and del Giorgio, 2010), we presented a flow cytometric protocol that allows not only the enumeration of HNF in natural water samples, but also the identification of distinct HNF cytometric populations based on their light scattering properties. More importantly, this method, which is based on the accumulation of the acidotropic fluorescent probe LysoTracker in HNF lysosomes (Rose et al., 2004), yields levels of digestive (feeding) activity of these cytometric populations, and therefore, allows to track the feeding responses of different HNF functional groups within mixed communities exposed to complex bacterial assemblages. In turn, the physiological structure of these complex bacterial communities can be described using flow cytometry and fluorescent markers that target different aspects of bacterial single-cell activity and composition (Gasol and del Giorgio, 2000). Here we combine these approaches to explore the potential interactions between single-cell digestive activity of mixed estuarine HNF communities, and the physiological structure of their bacterial prey community. We explored this question in re-growth cultures using natural estuarine bacterial and HNF communities, where we followed both HNF activity and bacterial abundance and physiologic structure through the different phases of the predator/prey dynamics that develop in these cultures through time.

## MATERIALS AND METHODS

### EXPERIMENTAL SETUP

The experimental approach consisted of following HNF and bacterial abundance, cytometric characteristics, and enzymatic activity in re-growth cultures of natural planktonic assemblages, as described previously (Sintes and del Giorgio, 2010). Briefly, four dilution cultures with natural bacterial communities and flagellates from the Choptank River, a subestuary of the Chesapeake Bay (Baltimore, MD, USA), were established by adding 100 ml of 2 μm filtered water (polycarbonate, Nuclepore) to 1 l of 0.2 μm-filtered (polycarbonate, Nuclepore) and heat-sterilized water from the same location. In this study, we monitor two of the four cultures used in our previous study, where we not only followed the abundance of bacterial cells, but also monitored their physiological structure to expand the analysis on their relationship to the HNF single-cell characteristics. The two cultures were incubated in the dark at room temperature and the dynamics of bacterial and HNF abundance and activity were monitored over 200 h as described below. The physiologic structure of bacterial communities is complex and multifaceted, and includes properties such as the cell size distribution, and the distribution of cells in various physiologic categories, such as highly active, living, dormant, injured and dead, which in turn can be quantified using a variety of probes (del Giorgio and Gasol, 2008). For simplicity, here we targeted three specific aspects of this structure, that have been previously shown to be linked to HNF grazing: the cells with high metabolic (respiratory) activity, detected using the intracellular reduction of the fluorescent tetrazolium salt CTC (del Giorgio et al., 1997), the proportion of cells with high and low DNA contents, detected by combining a fluorescent nucleic acid probe and light scatter using flow cytometry (Gasol et al., 1999), and the cells with a damaged membrane, detected using the combination of a stain that only penetrates cells with compromised membranes (Propidium Iodide) and a stain that penetrates all cells (Sachidanandham et al., 2005). However, further research should assess a wider set of physiological aspects of both bacteria and HNF.

### DETERMINATION OF HETEROTROPHIC BACTERIAL ABUNDANCE AND HIGH/LOW DNA CELLS

Heterotrophic bacterial abundance was determined on glutaraldehyde-fixed samples by flow cytometry using a FACSCalibur Flow Cytometer (Becton Dickinson). Samples (0.5 ml) were stained with SYTO 13 (2.5 mM final concentration, held at room temperature in the dark for 10 min) and 1 μm green fluorescent beads were added as an internal standard (del Giorgio et al., 1996). Finally, the samples were run at low flow through the flow cytometer and green fluorescence (FL1) and 90° light scatter (SSC) intensity recorded. Two populations were discriminated based on their signature in the FL1 vs. SSC cytogram: HNA (high nucleic acid content) and LNA (low nucleic acid content) bacteria.

### BACTERIAL SINGLE-CELL CHARACTERISTICS: ACTIVELY RESPIRING CELLS AND CELLS WITH DAMAGED OR COMPROMISED MEMBRANES

Highly active (respiring) cells were detected cytometrically using the reduction of CTC (del Giorgio et al., 1997). A stock solution of 50 mM CTC (Polysciences, Warrington, PA, USA) was prepared daily, filtered through 0.1 μm pore-size polycarbonate filters (Nuclepore) and kept dark at 5°C until use. A volume of 0.1 ml of CTC stock solution was added to 0.9 ml water samples resulting in a final CTC concentration of 5 mM in the samples and then incubated at room temperature in the dark for 2 h (del Giorgio et al., 1997). At the end of the incubation, 1 μm beads were added as internal standard and subsequently, the sample was run in the cytometer. The orange fluorescence of CTC (FL2) and the light side scatter emission were used to discriminate the CTC+ cells from CTC- cells and other particles. The percentage of CTC+ cells was calculated in relation to total bacterial counts, obtained by SYTO-13 staining.

The commercial LIVE/DEAD BacLight kit (Molecular Probes) was used in the two cultures to quantify cells with damaged or compromised cellular membranes. This kit contains a mixture of a cell-impermeant nucleic acid stain (Propidium Iodide) that only penetrates cells with damaged membranes, and a cell-permeant nucleic acid stain (SYTO-9) which acts as a counterstain for all cells. Three μl of the mixture of the two stains were added to 1 ml sample and subsequently, the samples were incubated at room temperature in the dark for 10 min. Then, 1 μm beads were added as internal standard and the samples were run at low flow in the cytometer. Cells with compromised membranes were discriminated from cells with intact membranes in a cytogram of red (FL3) vs. green (FL1) fluorescence (Sachidanandham et al., 2005; Berney et al., 2007).

### BETA-D-GLUCOSAMINIDASE ACTIVITY OF HNF

The beta-D-glucosaminidase enzymatic activity of the HNF was measured in duplicate once per day in each of the dilution cultures as previously described (Sintes and del Giorgio, 2010). Briefly, particulate enzyme activity (PEA) was measured as activity retained on a Whatman GF/F filter. Twenty-five to 50 ml of sample was filtered through a GF/F filter that was then placed in a vial containing 4 ml of the same GF/F filtered sample and 4 ml of reaction solution [0.05 M acetic buffer, pH 4.6, and 0.2% (w/v) 23 lauryl ether (Brij 35)]. After adding the reagents to the samples, each tube was sonicated at 50 W twice for 10 s. Finally, the fluorogenic substrate 4-methylumbelliferyl-*n*-acetyl-beta-D-glucosaminide was added at a final concentration of 20 mM. The controls consisted of the same samples processed as the others but without substrate added at the start of the incubation. The treatment and control vials were incubated in the dark at room temperature, and 1.5 ml aliquots were taken from each tube at time 0 and after 1, 6, and 24 h of incubation. These aliquots were placed in 7 ml glass vials containing 3 ml of 0.2 M CAPS, pH 10.3 to terminate the reaction (Sherr and Sherr, 1999), and substrate was then added to the controls. The fixed samples were stored in the dark at 2°C until measurement using a Shimadzu spectrofluorometer, within <2 d. Enzymatic activity in the samples was calculated using the slope of a standard curve made with known amounts of the product of the enzymatic reaction, 4-MUF, versus fluorescence. The enzyme activity obtained from the filtered water was subtracted from the filter+water activity to estimate the PEA. The cell-specific enzyme activity of HNF was calculated as the PEA divided by the abundance of HNF at the corresponding time point.

### FLOW CYTOMETRIC ANALYSES OF HNF

Heterotrophic nanoflagellate abundance and single-cell characteristics were analyzed by flow cytometry as described previously (Sintes and del Giorgio, 2010). Briefly, duplicate 2 ml water samples from each dilution cultures were stained with LysoTracker Green DND-26 (Molecular Probes) at a final concentration of 50 nM, and run in the flow cytometer at high flow rate (100 μl min^-1^) within the first 10 min after staining. Fluorescent beads (2.5 μm in diameter, Polysciences) were added at a final concentration of 7500 ml^-1^ to each sample as an internal standard. The values obtained for the duplicate determinations per sample were averaged. The FL1 and 90° light scatter (SSC) intensities were recorded. Up to three flagellate populations were discriminated in the FL1 versus SSC plot (Sintes and del Giorgio, 2010), subsequently termed small, medium, and large HNF.

The average FL1 of HNF cells associated to LysoTracker staining is significantly positively related to the specific activity of HNF derived from Beta-D-glucosaminidase (β-Gam) activity measurements (Sintes and del Giorgio, 2010). β-Gam activity is directly related to grazing activity (Vrba et al., 1996), consequently the relationship between FL1 associated to Lysotracker and the activity of β-Gam provides an index of the activity of digestive enzymes. We therefore used the average FL1 as an indication of the grazing activity of HNF (Sintes and del Giorgio, 2010), while the SSC was used as indication of cell morphology (size, internal structure, and membrane characteristics). The total HNF abundance represents the sum of the abundances of the various populations that could be discriminated in the cytograms.

## RESULTS

### DYNAMICS OF THE HETEROTROPHIC BACTERIAL AND NANOFLAGELLATE ABUNDANCE

**FIGURE 1:**
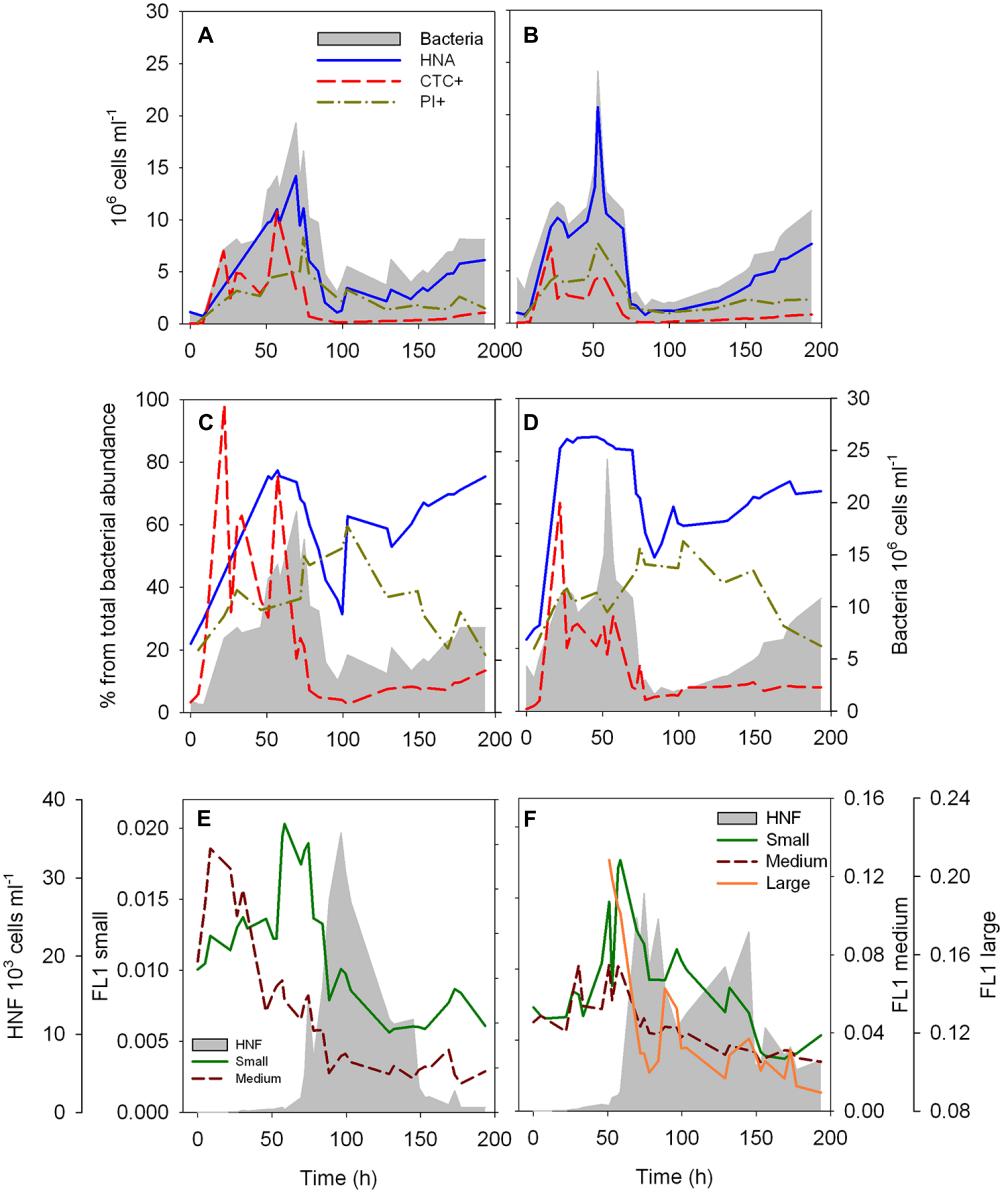
**Dynamics of the abundance **(A,B)** and proportion **(C,D)** of HNA, CTC+ and membrane compromised (PI+) cells over the time course of the two re-growth cultures, and dynamics of the fluorescence characteristics of the different HNF populations over the time course of the two re-growth cultures **(E,F)**.** The intensity of the green fluorescence (FL1) associated to LysoTracker Green of each HNF population is used as a proxy of single-cell digestive activity. Total bacterial and total HNF abundance (gray area) are plotted as a reference (Data of total bacterial and HNF abundance for the culture 2, **B,D,F**, was previously presented in Sintes and del Giorgio, 2010).

**Figure A1:**
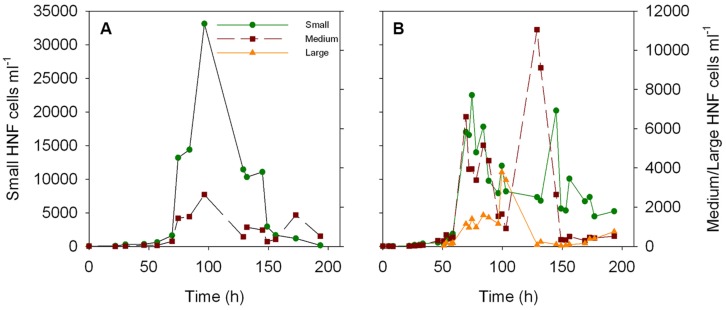
**Dynamics of the abundance of small, medium, and large HNF cells over the time course of the two re-growth cultures (A,B).** Data from culture 2 was previously presented in Figure 9 from Sintes and del Giorgio (2010).

The dynamics of the bacterial and HNF abundance in these re-growth cultures has been previously described in Sintes and del Giorgio (2010). Briefly, bacterial abundance increased over the first 40–70 h of the two re-growth cultures presented here (**Figures 1A,B**), thereafter decreasing steeply coinciding with an increase in HNF abundance (**Figures 1E,F**). Total HNF increased sharply at around 70 h (**Figures 1E,F**), remained at high abundance (>2 × 10^4^ cells ml^-1^) for ∼15 h and decreased afterward. A second peak in abundance was observed after 145 h of the start in one of the cultures (**Figure 1F**). The cytometric analysis allowed the identification of three, well-defined cytometric populations of HNF, which revealed that the abundance of small and medium HNF followed similar dynamics to the total HNF abundance, whereas the large HNF (only present in the second culture, **Figure 1F**) increased at around 100 h, coinciding with the lowest abundance of the other two HNF populations [**Figure A1 in Appendix**, see Figure 9A in Sintes and del Giorgio (2010)].

### DYNAMICS OF HNF FEEDING ACTIVITY AND BACTERIAL PHYSIOLOGIC STRUCTURE

The dynamics of the different cytometric populations of bacteria were roughly comparable between the two re-growth cultures. The abundance of HNA cells follows closely the total bacterial abundance (**Figures 1A,B**). The proportion of HNA cells increased rapidly in the first 20 h of the experiments (**Figures 1C,D**), and remained high (around 80% of total bacterial abundance) until ∼70 h. Subsequently, the %HNA cells decreased to 33–50%, and steadily increased again after 84–100 h. The abundance (**Figures 1A,B**) and proportion (**Figures 1C,D**) of HNA had similar dynamics over the course of the re-growth cultures. The abundance and proportion of CTC+ cells reached their maximum at 22 h, and %CTC+ remained above 20% of the bacterial community until 57 h, abruptly decreasing afterward and remaining below 10% of the total bacterial abundance for the remainder of the time course (**Figures 1C,D**). The proportion of cells with a damaged membrane steadily increased up to 55–60% at 103 h, followed by a decrease to 20% at the end of the experiments (**Figures 1C,D**), with a peak damaged cell abundance occurring after the peak in total bacterial abundance (**Figures 1A,B**).

The average FL1 associated to LysoTracker of the different HNF cytometric populations increased sharply and reached highest values at around 60 h in both cultures preceding the abundance peak of the HNF (**Figures 1E,F**). Exceptionally, the FL1 from the medium HNF from the first re-growth peaked already after 8.5–22 h of the start of the experiment (**Figure 1E**). A second, albeit smaller increase in FL1 of the different cytometric populations was detected before the second HNF abundance peak in the second culture (**Figure 1F**) and coincided with the increase in the proportion of HNA cells in both re-growth cultures (**Figures 1E,F**).

### RELATIONSHIPS BETWEEN BACTERIAL PHYSIOLOGICAL STRUCTURE AND FLAGELLATE ABUNDANCE

**FIGURE 2:**
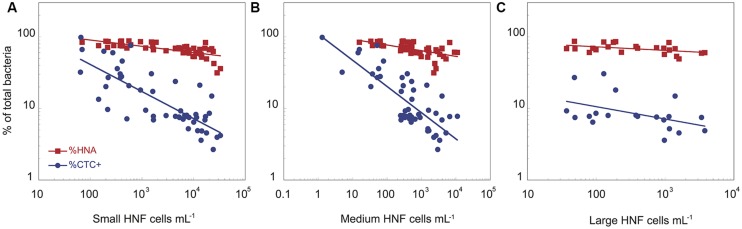
**The relationships between the abundance of small **(A)**, medium **(B)**, and large **(C)** heterotrophic flagellates and the proportion of HNA and CTC+ bacterial cells for the two cultures.** The data have been log-transformed.

The physiological structure of the bacterial community, in terms of the abundance and proportion of HNA and CTC+, correlated with the abundance of the different HNF cytometric populations (**Figure 2**). There was a negative relationship between the abundance of small, medium, and large HNF populations and both the proportion of HNA (%HNA = 135 × Small_HNF^-0.09^, *r*^2^ = 0.49, *p* < 0.001; %HNA = 111 × Medium_HNF^-0.08^, *r*^2^ = 0.37, *p* < 0.001; %HNA = 92 × Large_HNF^-0.05^, *r*^2^ = 0.19, *p* < 0.03) and the proportion of CTC+ cells (%CTC+ = 235 × Small_HNF^-0.38^, *r*^2^ = 0.53, *p* < 0.001; %CTC+ = 109 × Medium_HNF^-0.37^, *r*^2^ = 0.67, *p* < 0.001; %CTC+ = 23 × Large_HNF^-0.17^, *r*^2^ = 0.15, *p* < 0.05; **Figure 2**). In contrast, the proportion of cells with damaged membranes was positively related with the abundance of small and medium HNF (%PI+ = 21 × Small_HNF^0.08^, *r*^2^ = 0.38, *p* < 0.003, %PI+ = 25 × Medium_HNF^0.06^, *r*^2^ = 0.20, *p* < 0.01).

**FIGURE 3:**
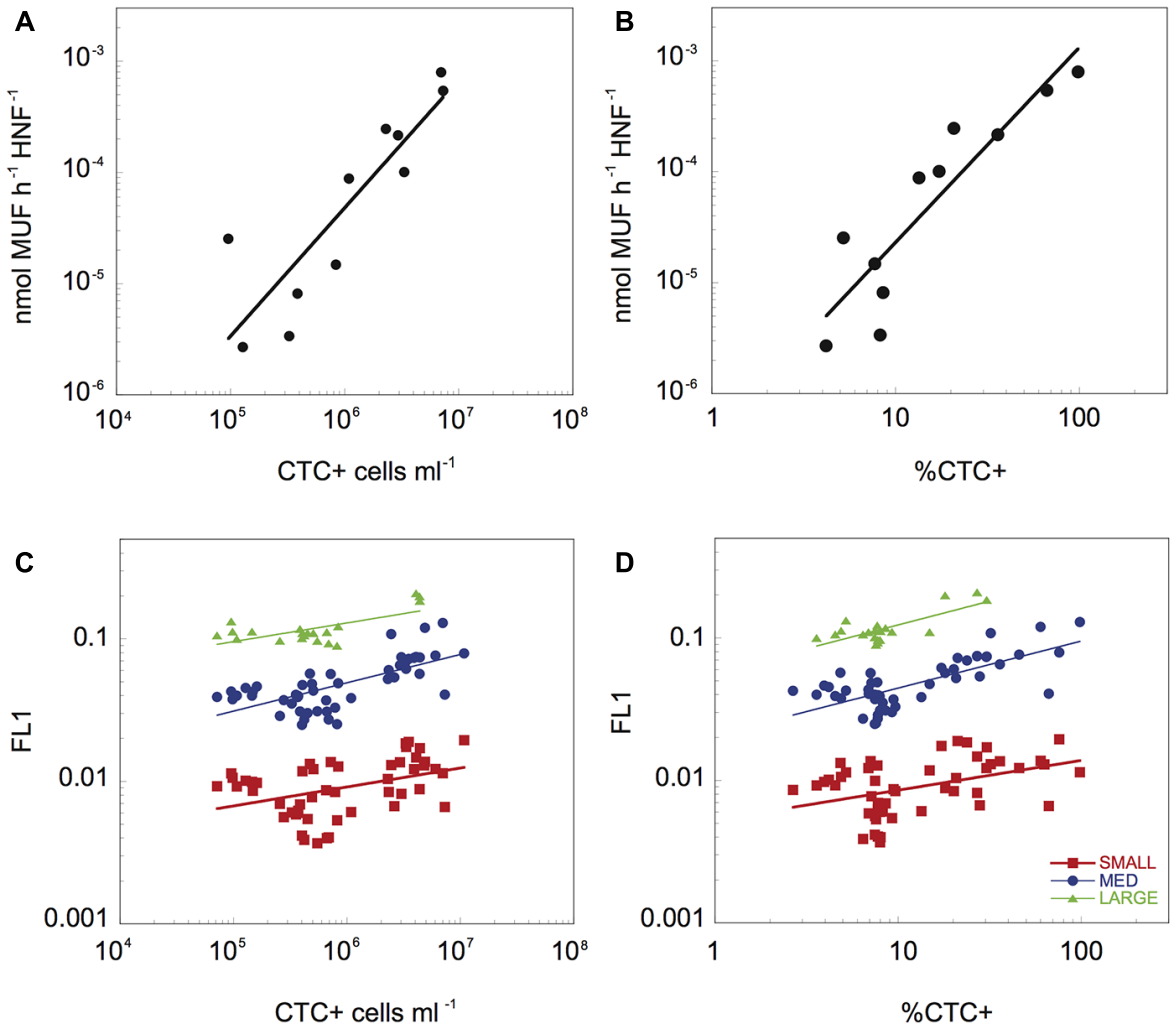
**The relationship between β-Gam activity per HNF cell and the abundance **(A)** and proportion of CTC+ cells **(B)**, and relationship between the average green fluorescence (FL1) associated to the three different HNF populations and the abundance **(C)** and proportion **(D)** of CTC+ cells.** The data have been log-transformed.

Heterotrophic nanoflagellate feeding activity, both at the bulk community level in terms of average specific β-D-glucosaminidase activity, as well as with the average FL1 of the different cytometric HNF populations (**Figure 3**) was significantly related to the physiological structure of the bacterial community. To avoid potential bias due to potential contribution of bacteria to the PEA, we have excluded the initial time points (up to 22 h) where bacterial biomass peaked and HNF biomass was still low. There was a strong positive relationship between bulk, cell-specific HNF enzymatic activity, with both the abundance of CTC+ cells (Cell-specific β-Gam = 5.32 10^-6^ × CTC+^1.15^, *r*^2^ = 0.89, *p* = 0.002, **Figure 3A**) and the percentage of CTC+ bacterial cells (Cell-specific β-Gam = 4.04 10^-7^ × %CTC+^1.76^, *r*^2^ = 0.94, *p* < 0.001, **Figure 3B**). Likewise, the feeding activity of the three main HNF cytometric populations, as reflected by their average FL1 values, was positively correlated to both the abundance of CTC+ cells (FL1-Large_HNF = 0.021 × CTC+^0.13^, *r*^2^ = 0.56, *p* < 0.002, FL1-Medium_HNF = 0.003 × CTC+^0.20^, *r*^2^ = 0.50, *p* < 0.001, and FL1-Small_HNF = 0.001 × CTC+^0.13^, *r*^2^ = 0.25, *p* < 0.003, **Figure 3C**), and to the proportion of CTC+ cells (FL1-Large_HNF = 0.057 × %CTC^0.33^, *r*^2^ = 0.66, *p* < 0.001, FL1-Medium_HNF = 0.021 × %CTC^0.33^, *r*^2^ = 0.60, *p* < 0.001, and FL1-Small_HNF = 0.005 × %CTC^0.21^, *r*^2^ = 0.21, *p* < 0.003, **Figure 3D**). The relationship between FL1 and the proportion HNA cells was in general weaker than that for %CTC, and was only significant for the large and medium HNF (*r*^2^ = 0.42, *p* < 0.005, and *r*^2^ = 0.07, *p* = 0.03, respectively, data not shown). The proportion of cells with compromised membrane only correlated significantly with the FL1 of small_HNF (*r^2^* = 0.25, *p* = 0.02).

## DISCUSSION

The flagellate-bacterial dynamics that we observed here (**Figure 1**) are in accordance with previous observations that HNF abundance strongly influences bacterial abundance (Sanders et al., 1992), and suggest a strong grazing control of bacteria during these re-growth experiments (Gasol and Vaque, 1993). Our results further suggest that HNF preferentially grazed on CTC+ cells, in agreement with previous reports (del Giorgio et al., 1996), and to a lesser extent, on HNA cells (**Figure 2**), perhaps reflecting the weaker connection to cellular activity that exists for the latter groups (Bouvier et al., 2007). Our results thus confirm that HNF predators influence not only the total bacterial abundance, but also the size distribution and physiological structure of bacterioplankton communities (Andersson et al., 1986; Hahn and Hofle, 1998; Corno et al., 2008; del Giorgio and Gasol, 2008), and it has been proposed that this selective cropping of larger, faster-growing bacteria may cause further shifts in bacterial community composition (Simek et al., 2001; Pernthaler, 2005) and function, for example, in terms of substrate-utilization profiles (Corno and Jurgens, 2008). It is interesting to note that the number and proportion of cells with damaged membranes peaked after the maxima of HNF feeding activity, suggesting a connection between HNF grazing and bacterial cell injury, perhaps through incomplete digestion of ingested cells.

Perhaps more importantly to the objectives of this study, our results suggest that there is a strong reciprocal link between HNF single-cell activity and bacterial physiologic structure, such that HNF grazing determines the abundance and the proportion of highly active and growing bacterial cells (**Figure 2**), which in turn influence the level of the HNF feeding activity (**Figure 3**). Culture studies have shown that flagellates can rapidly resume growth after starvation, and undergo morphological and physiological changes such as increased O_2_ consumption and variations in mitochondrial volume when prey become available (Fenchel, 1982b). HNF have been also shown to have differential lag phases and growth responses when fed on different bacterial strains (Simek et al., 2013), suggesting a strong influence of prey quality. Our results suggest that this is also the case for mixed HNF assemblages feeding of natural bacterial prey, and further suggest that this rapid response involves large and rapid shifts in digestive enzymatic activity in response to variations in preferred prey availability. Cell-specific β-Gam activity is related to grazing and to overall cell metabolic activity of HNF cells (Bakalara et al., 1995; Minami et al., 2003), and here we have shown a positive relationship between the cell-specific enzyme activity, based on bulk enzymatic measurements and total HNF density, with both the abundance and the relative proportion of CTC+ cells. In a previous study we had reported a strong relationship between this enzymatic activity and the average green single-cell fluorescence (FL1) using LysoTracker (Sintes and del Giorgio, 2010), and the application of the cytometric approach here further allowed us to resolve shifts in the digestive activity at the single-cell level of the various cytometric populations of HNF that developed in the re-growth cultures. In all three HNF populations identified using flow cytometry we observed a variation in cell-specific digestive activity of at least one order of magnitude, and in all cases, this variability was linked to the dynamics of CTC+ cells (**Figures 3C,D**). Remarkably, the strength of this correlation was different for the different HNF cytometric populations, and was strongest for the large HNF, relative to the small and medium HNF, suggesting differential sensitivity of these different HNF cytometric populations to the physiological structure of the prey bacterial communities. Protist cell size has major implications for their physiology and energetics. Interestingly, the large HNF in the re-growth cultures had higher cell-specific digestive activity (FL1 = 0.124 ± 0.035) than the medium (0.055 ± 0.027) and the small (0.010 ± 0.004) HNF, in agreement with reported higher growth and grazing rates of larger HNF (Fenchel, 1982a; Choi and Peters, 1992). The higher growth and grazing rates supports the faster/stronger response of the large HNF to the presence of the desired prey. However, further studies with different sized HNF populations will expectably add up information to the degree of correlation between the cell-specific activity and %CTC+ cells.

As we pointed out in our previous paper (Sintes and del Giorgio, 2010), it is not clear whether the HNF cytometric populations described here corresponded to a single HNF population or to several populations overlapping in their cytometric signatures. In the context of our study, the coherent behavior of these the HNF cytometric populations suggests that they can be considered as functional units, and that the major shifts in cytometric parameters are due to consistent shifts in cell properties within the consortium (Sintes and del Giorgio, 2010). Thus, the response of the HNF cytometric populations described here corresponds to the average response of a mixed community of HNF within each functional unit. These mixed HNF functional groups that were identified in the dilution cultures yield in our opinion a more realistic representation of the functioning of ambient mixed communities than the experimental use of individual taxa, but future work should focus on elucidating the composition of these functional groups, and the potential species-specific responses within them, for example using cell sorting (Vazquez-Dominguez et al., 2005).

It is interesting to note that HNF abundance was positively related to the absolute abundance of active (CTC+) bacteria (*r*^2^ = 0.50, *p* = 0.02), and even more pronouncedly to the proportion of CTC cells (*r*^2^ = 0.93, *p* < 0.001), suggesting that HNF communities respond numerically to large shifts in active bacterial cell abundance, but respond also metabolically to more subtle shifts in the relative availability of target prey; it is thus the interplay between the two that determines the total grazing activity. The different intensity of the response to abundance and proportion of preferred prey cells could indicate that in addition to their absolute abundance, the dilution of suitable prey within the total bacterial community may play a role in shaping encounter rates and therefore grazing activity.

There has been extensive work on the response of HNF to shifts in prey quality and availability, and most of these studies have demonstrated a remarkable versatility in the functional response of these protists (Choi, 1993; Cleven and Weisse, 2001; Boenigk and Arndt, 2002; Montagnes et al., 2008). The majority of these studies, however, have focused on bulk community level measures of activity (Montagnes et al., 2008; Jürgens and Massana, 2008), and our study is among the first to quantify shifts in single-cell grazing and metabolic activity as a response to variations in prey availability. More importantly, preferred prey availability, both in terms of their absolute cell abundance as well as the relative proportion of CTC+ cells, was itself determined by grazing, thus establishing a tight negative feedback loop at the single-cell level. Whereas this type of feedback has been shown for other predator-prey systems, e.g., worms predating on ciliates (Hammill et al., 2010), to the best of our knowledge, this is the first time that the functional response of natural HNF assemblages has been shown to be induced by a change in the physiologic structure of their mixed bacterial prey.

This feedback at the single-cell level that we report here has profound ecological implications. Bacterioplankton abundance in natural environments is remarkable constant both across and within aquatic ecosystems, in spite of their remarkable potential for growth, and of the highly variable and heterogeneous environments in which they live (del Giorgio and Gasol, 2008). This relative invariance in bacterial abundance has been explained in part by tight numerical coupling between bacteria and their protistan grazers, which can potentially grow at similar rates (Fenchel, 1982a,c), and although some studies have reported the typical predator-prey oscillations in bacterial and HNF abundance in ambient waters (Andersen and Sorensen, 1986), most studies report either little or no co-variation in bacterial and HNF density (Gasol and Vaque, 1993). However, predator-prey oscillations frequently occur in dilution cultures such as the ones used in this study (Beardsley et al., 2003). These findings have been explained by the rapid growth of some specific bacterial taxa induced by the reduced grazing pressure in dilution cultures. These specific bacterial taxa grow larger than other bacterioplankton cells and are grazed preferably during the growth of the flagellates (Pernthaler, 2005).

The proposed interactions may result from rapid eco-evolution of both prey and predator (Yoshida et al., 2003; Jones et al., 2009), in which prey and predator populations alter their environment (e.g., through predation, nutrient release), and these changes in the environment in turn influence the subsequent evolution of the population (Post and Palkovacs, 2009). The eco-evolutionary dynamics within communities can lead to unusual predator-prey cycles, such as nearly anti-phase predator-prey cycles like the cycles observed in this study, i.e., ∼50% lag phase, in contrast to the “classic” predator-prey cycle that would have roughly a quarter cycle delay (Yoshida et al., 2003). These unusual cycles may take place when rapid evolution of prey occurs as a consequence of competition between prey species or clones (Yoshida et al., 2003), when there is alternation of functional traits through alternation in species density and compensatory dynamics between functionally different species (Tirok and Gaedke, 2010), or when both predator and prey can change their phenotypes through adaptive plasticity (inducible defenses and offenses; Yoshida et al., 2007; Mougi, 2012). In this regard, Mougi (2012) proposed that in a predator-prey system where both actors exhibit phenotypic plasticity, in terms of morphologcial and physiological adjustments, this plasticity will tend to maximize their fitness but may result in unusual dynamics, especially when the carrying capacity of the prey is small (Mougi, 2012). In our study, the change in the proportion of preferred cells (reduction in CTC+ cells) could be analogous to a defensive trait, as HNF selectively feeds on active cells, and thus could be comparable to inducible defenses, and the change in the single-cell activity of the HNF might be assimilated to an inducible offense, thus supporting the theoretical model of Mougi (2012).

**FIGURE 4:**
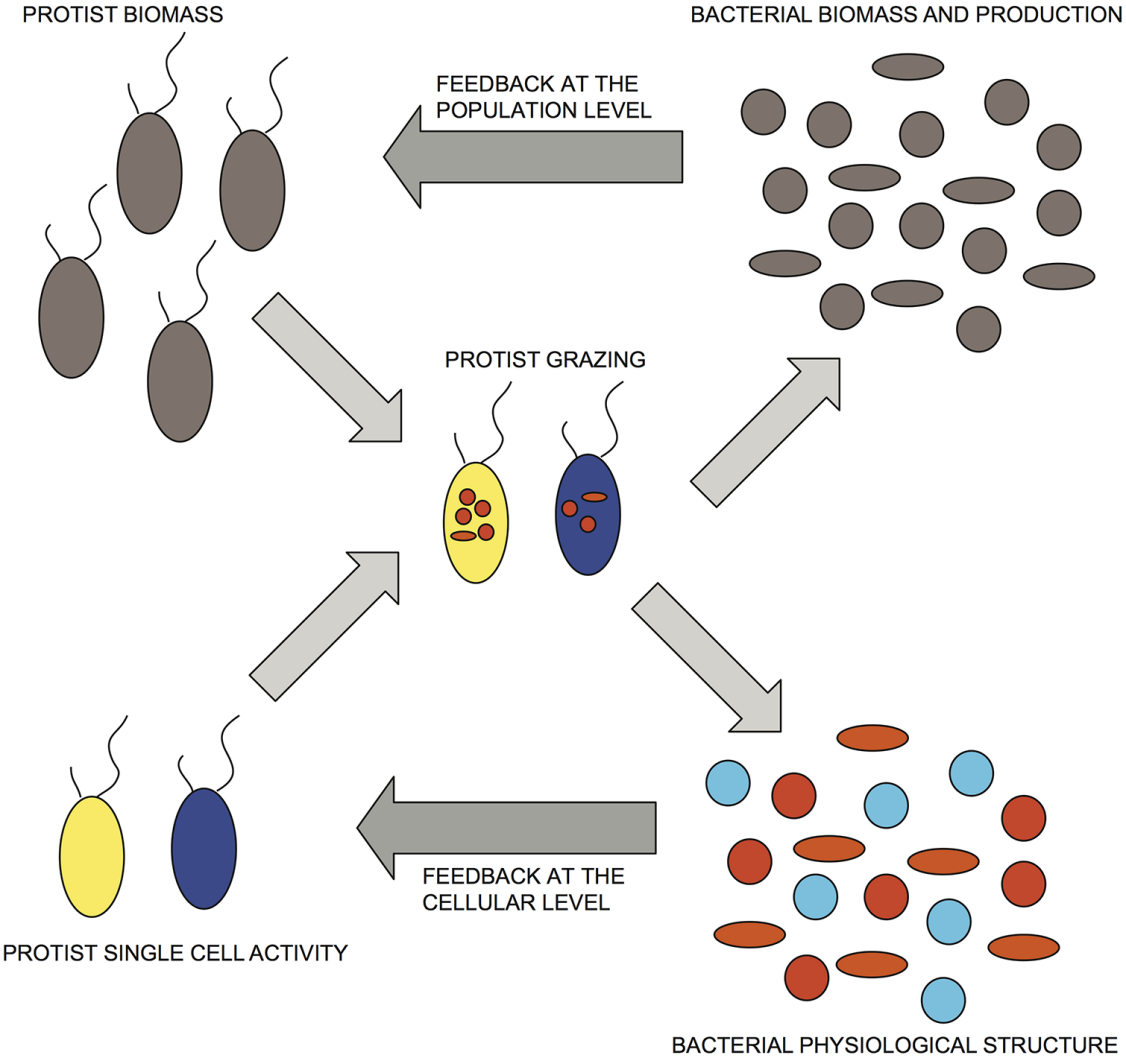
**Conceptual representation of bacterial/HNF interactions that incorporate both numerical and functional responses.** Dark-gray arrows indicate proposed feedbacks at the population and cellular levels. Bacterial physiological structure, cell activity: red > orange > blue; protist single-cell activity: yellow > blue.

Based on these results, we propose a conceptual model that is an extension of current accepted models of aquatic bacterial/HNF interactions, wherein the individual grazing activity of HNF is finely tuned not only to the total bacterial abundance but to the relative distribution of preferred prey, corresponding in our study to highly active and growing cells (**Figure 4**), such that there can be rapid shifts in HNF activity as a response to changes in the abundance and relative proportion of these more desirable prey (**Figure 4**). This scheme implies potentially large and rapid downshifts in total grazing rates as a feedback response to decreases in the abundance and proportion of highly active cells (**Figure 3**), that are not necessarily mediated by changes in HNF abundance. The numerical response would be superimposed to this negative control loop based on up- or downshifts in HNF cell activity, but this numerical response would be triggered only by larger shifts in bacterial abundance induced by environmental or other factors. The shift in specific cellular activity occurs at much shorter time scales than population level shifts in flagellate abundance, and offers a mechanism to explain both the apparent lack of coupling between bacterial an HNF abundances *in situ*, and also the relative constancy of aquatic bacterial abundance in time and space in natural aquatic environments.

## Conflict of Interest Statement

The authors declare that the research was conducted in the absence of any commercial or financial relationships that could be construed as a potential conflict of interest.
